# The epidemiology of *Helicobacter* *pylori* resistance in a university affiliated hospital: a comparison between two time periods—a cross-sectional design

**DOI:** 10.1186/s40001-023-01500-0

**Published:** 2023-11-24

**Authors:** Haim Shmuely, Yekaterina Shvartsman, Rita Berdinstein, Jacob Yahav, Ehud Melzer

**Affiliations:** 1 Department of Internal Medicine D, and Helicobacter Research Institute, Kaplan Medical Center, Rehovot, Israel; 2 Microbiology Laboratory, Kaplan Medical Center, Rehovot, Israel; 3 Faculty of Medicine, Hebrew University, Jerusalem, Israel; 4 Department of Gastroenterology, Kaplan Medical Center, Rehovot, Israel

**Keywords:** *Helicobacter pylori*, Antimicrobial resistance, Single hospital, Predictors for resistance

## Abstract

**Purpose:**

During the last few decades, the increased use of various types of antibiotics in the general population caused a significant change in regional *Helicobacter pylori* (*H. pylori)* antibiotic resistance. Our aim is to study the changes in *H. pylori* resistance in patients who had undergone an esophagogastroduodenoscopy (EGD) and susceptibility testing and found positive for *H. pylori.* The study was conducted in a university affiliated hospital between 2013–2020.

**Methods:**

A cross-sectional study was performed on all consecutive patients who had undergone an EGD and tested positive for *H. pylori* at the Kaplan Medical Center, Israel. The study period was divided into two sub-periods: 2013–2016 and 2017–2020. Data on age, sex, comorbidities, previous treatments, and antimicrobial susceptibility testing for six antimicrobial agents were compared.

**Results:**

The resistance rates of *H. pylori* to clarithromycin and dual resistance to clarithromycin and metronidazole were found significantly higher during the late period. Multivariable analysis showed that the later period, older age, and diabetes mellitus were independent predictors for antimicrobial resistance.

**Conclusions:**

Our study has shown that there is an increasing resistance of *H. pylori* to clarithromycin and metronidazole while its susceptibility is unaffected with time to other antibiotics. More recent cross-sectional studies with larger samples are warranted in order to evaluate the changes in the resistance patterns of *H. pylori* to various antibiotics with time.

## Introduction

*Helicobacter pylori* (*H. pylori*) infection has been linked to peptic ulcer disease and gastric cancer [1]. The seroprevalence of *H. pylori* in Israel is ~ 45%; the annual incidence of gastric cancer is ~ 9/100,000 people [2, 3]. The first line of *H. pylori* treatment in Israel includes a proton pump inhibitor (PPI), amoxicillin (AMP), clarithromycin (CLR) and/or metronidazole (MET); the second line comprises PPI, and bismuth quadruple therapy, including tetracycline (TET) and MET. Triple therapy comprises PPI, AMP and levofloxacin (LEV) or rifabutin (RIF) which is used as a rescue therapy. Resistance prevalence varies by country and within the same region. Possible causes of treatment failure are the increased use of different antibiotics prescribed for adults and children for dental work, and respiratory, gynecological, and parasitic infectious diseases. Primary resistance can significantly impair the efficacy of eradication regimens, especially the inclusion of macrolides (i.e., clarithromycin) [4]. High secondary resistance to metronidazole or clarithromycin is expected after treatment failure with therapeutic combinations containing one of these compounds [5]. Since multi-resistance frequently develops against most implemented antibiotics thus, leading to increased inadequate success rates, new therapeutic strategies are urgently needed. Only a few Israeli studies have investigated antibiotic resistance of *H. pylori* [6, 7].

The rates of antibiotic resistance may fluctuate over the years and between different regions [8]. The aims of this study were to evaluate recent changes in *H. pylori* resistance between patients who had undergone an EGD and susceptibility testing and were found positive for *H.* pylori, in addition to identifying independent predictors for antimicrobial resistance in a single hospital during 2013–2016 (the early period) and 2017–2020 (the later period).

## Methods

### Study design

Cross-sectional.

### Setting

The Department of Gastroenterology, Kaplan Medical Center, a university-affiliated hospital (600 beds) located in central Israel. Kaplan Medical Center’s institutional review board approved the study.

### Participants

Between January 1, 2013 and December 31, 2020, 269 clinical isolates of *H. pylori* were isolated from antral biopsy specimens of 269 adult patients, one isolate from each patient. Fifty-seven were naïve patients previously not treated for *H. pylori* infection; 212 had been previously treated for this infection and had failed ≥ 1 treatment regimen, i.e., first-line triple therapy with a PPI, AMP, CLR or MET or second-line quadruple therapy with second-line quadruple therapy with PPI, MET, TET and bismuth or rescue treatment with PPI, AMP, LEV or RIF. The study period was divided into two sub-periods, 2013 to 2016 and 2017 to 2020. Patient characteristics and antimicrobial resistance between the two periods were compared.

### Variables/data sources/measurement

Biopsy specimens were inoculated directly onto a Columbia blood agar (Difco, Detroit, MI) supplemented with a yeast extract of 5 g/L, laked lysed horse blood (7%), vancomycin (3 mg/L), colistin sulfate (7.5 mg/L), nystatin (12,500 IU/L) and co-trimoxazole (5 mg/L). Cultures were incubated for 72h at 37°C under microaerophilic conditions*. H. pylori* isolates were identified by colony morphology, characteristic spiral morphology on Gram staining and positive findings on catalase, urease, and oxidase tests. Susceptibility to six antibiotic agents: AMP, CLR, MET, TET, LEV and rifampicin were tested by the E-test (BioMérieux France). The strips were placed on dry agar plates. The minimum inhibitory concentration (MIC) values were determined after 72 h of incubation according to the E-test’s instructions. The *H. pylori* strain ATCC 43526 was used as quality control for the selective medium. The files of all cases were reviewed for age, gender, and comorbidities. Data regarding allergies to antibiotics and previous eradication treatments were identified from the patient's electronic medical records and recorded. Susceptibility to MET, CLR, TET, AMP, LEV and RIF was obtained from Kaplan’s Microbiology Laboratory electronic system.

### Quantitative variables

Resistance was defined according to the clinical breakpoints proposed by the European Committee on Antimicrobial Susceptibility Testing for *H. pylori* [9]: AMP, MIC > 0.125 mg/l; TET, MIC > 1 mg/l; CLR, MIC > 0.5 mg/l; MET, MIC > 8 mg/l, LEV, MIC > 1 mg/l and RIF, MIC > 1 mg/l.

### Statistical methods

Categorical variables were expressed as number and percentages. Distribution of age was assessed using a histogram and Q–Q plot. Since age was not normally distributed, it was reported as median and interquartile range (IQR). Categorical variables were compared by the Chi-square or Fisher's exact tests; age was compared by the Mann–Whitney test. Multivariable logistic regression calculated the independent association between time period and antimicrobial resistance. The time period was forced into the regression at the first block and then at the second. Variables that were significantly associated with the studied outcome were included in the regression. The backward method was applied for the second block using p > 0.1, the Wald test as criteria for variable removal. All statistical tests were two-sided; p < 0.05 was considered statistically significant. SPSS software was used for all statistical analyses (IBM SPSS statistics for window, version 27, IBM corp., Armonk, NY, USA, 2020).

## Results

### Patients

The study included 269 *H. pylori* isolates cultured from 269 patients. Of them, 86 were cultured before 2017 and 183 after. A comparison of the patients’ characteristics is presented in Table 1. Age, sex, comorbidities: diabetes mellitus (DM), ischemic heart disease, congestive heart failure and chronic renal failure were similar during the two periods. Patients grouped in the later period had a higher prevalence of DM (16.9% vs 7% *P* < 0.02). Allergies to penicillin, CLR, MET and TET were similar in the two groups. More previously treated patients and naïve untreated patients were included (*P* = 0.003) in the later period group; 135 had been previously treated for infection and failed more than one treatment regimen (first-line triple therapy with PPI, AMP, CLR, and/or MET or second-line quadruple therapy with second-line quadruple therapy with PPI, MET, TET, and bismuth or rescue treatment with PPI, AMP, LEV or RIF). Patients in the later period group were treated significantly more with CLR, MET, TET and bismuth.

**Table 1:** Comparison of patient's characteristics between the two periods

	2013–2016	2017–2020	*p*
	(*n* = 86)	(*n* = 183)	
Age	45 (30–59.3)	45.3 (32.6–61.5)	0.458
Gender—female	63 (73.3%)	125 (68.3%)	0.409
Comorbidities			
Diabetes	6 (7%)	31 (16.9%)	0.027
Ischemic heart disease	6 (7%)	15 (8.2%)	0.728
Congestive heart failure	2 (2.3%)	6 (3.3%)	> 0.999
Chronic renal failure	4 (4.7%)	3 (1.6%)	0.215
Allergy			
No allergy	74 (86%)	156 (85.2%)	0.968
Penicillin	12 (14%)	23 (12.6%)	
Clarithromycin	0 (0%)	1 (0.5%)	
Tetracycline	0 (0%)	2 (1.1%)	
Penicillin and clarithromycin	0 (0%)	1 (0.5%)	
Previous antibiotics received			
Previously treated	77 (89.5%)	135 (73.8%)	0.003
Naïve not treated	9 (10.5%)	48 (26.2%)	
Antibiotic received			
Amoxicillin	63 (73.3%)	112 (61.2%)	0.053
Clarithromycin	75 (87.2%)	121 (66.1%)	< 0.001
Metronidazole	53 (61.6%)	89 (48.6%)	0.046
Tetracycline	45 (52.3%)	58 (31.7%)	0.001
Bismuth	44 (51.2%)	56 (30.6%)	0.001
Levofloxacin	15 (17.4%)	24 (13.1%)	0.374
Rifabutin	1 (1.2%)	2 (1.1%)	> 0.999

### Culture and susceptibility

One hundred and thirty (71%) isolates out of 183 isolates of *H. pylori* were found significantly more resistant to CLR during the later period compared to 42 out of 86 isolates (48.8%, P < 0.001) during the early period (Table 2). Significantly higher dual CLR + MET resistance was seen during the late period in 59.6% isolates compared to 40.7% during the early period (P = 0.004). No statistically significant difference was found in resistance to MET, LEV or in simultaneous resistance for CLR-MET-LEV between the 2 time periods (Table 1). All cultures tested for AMP, TET and rifampicin in both periods, showed antibiotic susceptibility.

**Table 2:** Comparison of antibiotic resistance of *Helicobacter pylori* isolates between the two periods

	Early period 2013–2016	Late period 2017–2020	*p*
CLR resistance	42 (48.8%)	130 (71%)	< 0.001
MET resistance	60 (69.8%)	135 (73.8%)	0.493
LEV resistance	2 (2.3%)	1 (0.5%)	0.240
CLR or MET resistance	67 (77.9%)	156 (85.2%)	0.136
CLR + MET resistance	35 (40.7%)	109 (59.6%)	0.004
CLR + MET + LEV resistance	1 (1.2%)	1 (0.5%)	0.240

CLR, clarithromycin; MET, metronidazole; LEV, levofloxacin

### Factors independently associated with antibiotic resistance

A multivariate regression analysis revealed that the late period was an independent predictor of resistance to CLR (P = 0.001) and CLR + MET (P = 0.004) (Table 3). Increased age was significantly associated with resistance to CLR (P < 0.001), MET (P < 0.001) and dual resistance to CLR and MET (P < 0.001). DM was a predictor of MET resistance (P = 0.037). No statistically significant association was found between antibiotic resistance, gender, ischemic heart disease, congestive heart failure or previous antibiotic treatments.

**Table 3:** Factors independently associated with antibiotic resistance—a multivariate regression analysis

	CLR		MET		CLR + MET	
	Adjusted OR (95% CI)	*p*	Adjusted OR (95% CI)	*p*	Adjusted OR (95% CI)	*p*
Late period	2.64 (1.5–4.6)	0.001	1.21 (0.65–2.21)	0.562	2.37 (1.31–4.28)	0.004
Age	1.04 (1.02–1.06)	< 0.001	1.05 (1.03–1.08)	< 0.001	1.06 (1.03–1.08)	< 0.001
Male	1.37 (0.75–2.51)	0.336	1.37 (0.72–2.61)	0.336	1.16 (0.64–2.09)	0.627
DM	1.25 (0.43–3.58)	0.682	4.41 (1.09–17.82)	0.037	2.1 (0.76–5.8)	0.152
IHD	1.37 (0.25–7.48)	0.717	1.41 (0.16–12.68)	0.758	1.08 (0.23–4.97)	0.925
CHF	0.18 (0.02–1.82)	0.146	0.19 (0.01–2.78)	0.222	1.04 (0.14–7.91)	0.967
Previously treated	1.35 (0.65–2.82)	0.420	1.13 (0.5–2.55)	0.776	1.2 (0.58–2.46)	0.624

CLR, clarithromycin; MET, metronidazole; LEV, levofloxacin; DM, diabetes mellitus; HTN, hypertension; IHD, ischemic heart disease; CHD, congestive heart failure; CRF, chronic renal failure

## Discussion

In this study, we compared patients’ characteristics and changes in *H. pylori* resistance in those who had undergone an EGD, *H. pylori* with susceptibility testing and were found positive for *H. pylori*. The study took place in a university affiliated hospital between 2013 to 2016 and 2017 to 2020. The main finding of our study was the significant change in *H. pylori* resistance to CLR and CLR + MET observed during the late period (2017–2020). We found that *H. pylori* resistance to CLR and CLR + MET was significantly more common during the late period (71.0% vs 48.8%, P < 0.001 and 59.6% vs 40.7%, P = 0.004, respectively).

The CLR resistance rate has been increasing since 1998 [10], although, since 2008, to a lesser extent in Europe [11]. Antibiotic misuse is the primary cause of the increase in resistance. Exposure to antibiotics is very high, Valle Muñoz et al. [12] reported that up to 46% of the patients for whom an eradication treatment was indicated, had received macrolides (mainly, CLR and azithromycin) during the previous 12–14 years. Yet, in our study, previous antibiotic treatment was not associated with antibiotic resistance in the multivariate analysis. Out of 130 *H. pylori* resistant to CLR in later period, 83 (63.8%) were previously treated with CLR. Out of 109 *H. pylori* resistance to CLR + MET in later period, sixty-six (60.6%) were previously treated with CLR.

A correlation between antibiotic administration and resistance to CLR has recently been proven in Europe [11, 13]. The *H. pylori* rate of resistance to LEV during the later period was 13.1%, as new quinolones in Israel are restricted. Resistance to rifampicin was 1.1%, as the use of RIF is very low and mostly used to treat mycobacterial infections.

Univariate analysis showed that the patient groups in the early and later periods were of similar age (mean 45 years vs 45.3 years, P = 0.458) and sex (females: 73.3% vs 68.3%, P = 0.409). The patient population in the later period had a higher prevalence of diabetes.

We also found that the later period and older age were independent predictors of CLR resistance, most probably related to the excessive use of macrolides for respiratory tract infections worldwide, including Israel [13–16]. Older age was an independent predictor of resistance to CLR, MET and CLR + MET suggesting longer exposure to empiric antibiotic therapy with macrolides and MET for common infections throughout the years [17]. Double *H. pylori* resistance to CLR and MET has been associated with a later period in time and older age, probably relating to our finding that patients in the later period group were treated significantly more with CLR and MET.

In our study, DM was found to be an independent predictor of MET resistance. Higher MET resistance in *H. pylori*-infected DM patients may be a result of the frequent use of these antibiotics for recurrent anaerobic bacterial infections due to the development of MET-resistant strains [18, 19].

## Conclusions

Our study has shown that there is an increasing resistance of *H. pylori* to clarithromycin and metronidazole while its susceptibility is unaffected with time to other antibiotics. More recent cross-sectional studies with larger samples are warranted in order to evaluate the changes in the resistance patterns of *H. pylori* to various antibiotics with time.

## Data Availability

The datasets used and/or analyzed during the current study are available from the corresponding author on reasonable request.

## Abbreviations

MIC: Minimum inhibitory concentration
HTN: Hypertension
IHD: Ischemic heart disease
CHD: Congestive heart failure
CRF: Chronic renal failure
H. pylori: *Helicobacter pylori*
PPI: Proton pump inhibitor
AMP: Amoxicillin
CLR: Clarithromycin
MET: Metronidazole
TET: Tetracycline
LEV: Levofloxacin
RIF: Rifabutin
IQR: Median and interquartile range
DM: Diabetes mellitus
